# Structural evidence of the species-dependent albumin binding of the modified cyclic phosphatidic acid with cytotoxic properties

**DOI:** 10.1042/BSR20160089

**Published:** 2016-06-03

**Authors:** Bartosz Sekula, Anna Ciesielska, Przemyslaw Rytczak, Maria Koziołkiewicz, Anna Bujacz

**Affiliations:** * Institute of Technical Biochemistry, Faculty of Biotechnology and Food Sciences, Lodz University of Technology, Stefanowskiego 4/10, 90-924 Lodz, Poland

**Keywords:** antiproliferative action, crystal structure, cyclic phosphatidic acid (cPA), lysophospholipid transport, modified lysophospholipids, serum albumin

## Abstract

Cyclic phosphatidic acids (cPAs) are naturally occurring, very active signalling molecules, which are involved in several pathological states, such as cancer, diabetes or obesity. As molecules of highly lipidic character found in the circulatory system, cPAs are bound and transported by the main extracellular lipid binding protein–serum albumin. Here, we present the detailed interactions between human serum albumin (HSA) and equine serum albumin (ESA) with a derivative of cPA, 1-*O*-myristoyl-*sn*-glycerol-2,3-cyclic phosphorodithioate (Myr-2S-cPA). Initial selection of the ligand used for the structural study was made by the analysis of the therapeutically promising properties of the sulfur containing analogues of cPA in respect to the unmodified lysophospholipids (LPLs). Substitution of one or two non-bridging oxygen atoms in the phosphate group with one or two sulfur atoms increases the cytotoxic effect of cPAs up to 60% on the human prostate cancer (PC) cells. Myr-2S-cPA reduces cancer cell viability in a dose-dependent manner, with IC_50_ value of 29.0 μM after 24 h incubation, which is almost 30% lower than IC_50_ of single substituted phosphorothioate cPA. Although, the structural homology between HSA and ESA is big, their crystal complexes with Myr-2S-cPA demonstrate significantly different mode of binding of this LPL analogue. HSA binds three molecules of Myr-2S-cPA, whereas ESA only one. Moreover, none of the identified Myr-2S-cPA binding sites overlap in both albumins.

## Abstract (precis)

Cytotoxic properties of a new phosphorodithioate myristoyl derivative of cyclic phosphatidic acid as well as detailed binding mode of this ligand by human and equine serum albumins based on two crystal structures are presented.

## INTRODUCTION

**Figure 1:**
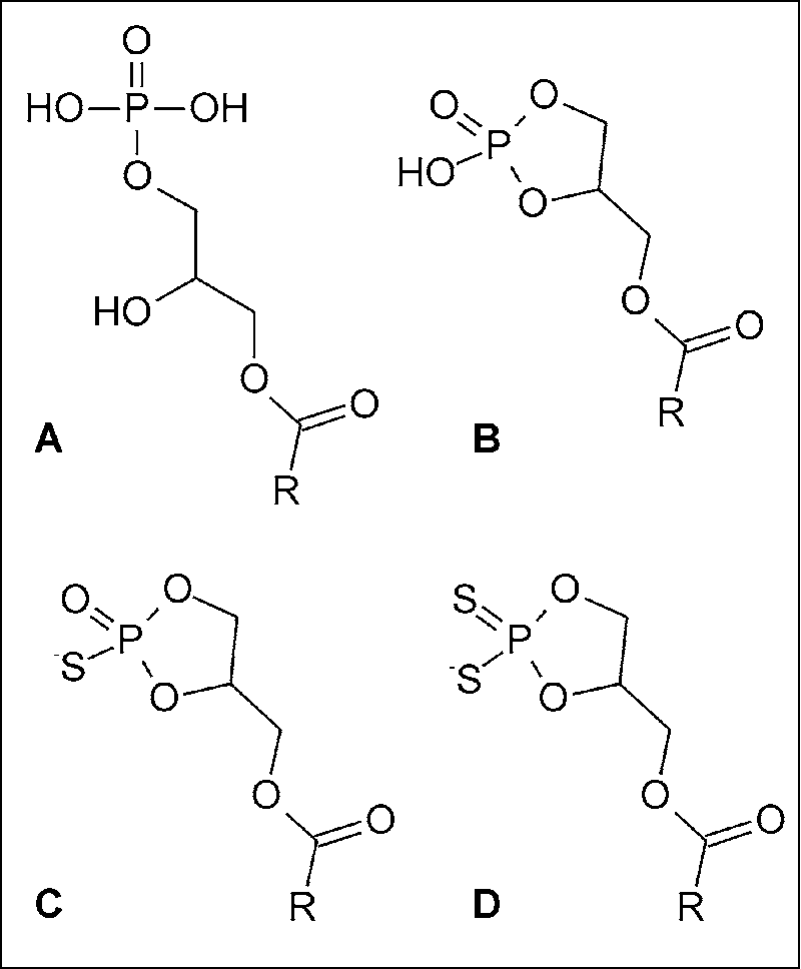
Chemical structures of studied lysophospholipids (**A**) Lysophosphatidic acid, (**B**) cyclic phosphatidic acid, (**C**) phosphorothioate and (**D**) phosphorodithioate analogues of cyclic phosphatidic acid. R indicates the aliphatic chain of fatty acid residue.

During the last two decades it has been shown that lysophospholipids (LPLs) are important signalling molecules involved in many physiological and pathophysiological processes [1]. So far, the best characterized LPLs are sphingosine-1-phosphate (S1P), lysophosphatidic acids (LPAs, Figure 1A) and cyclic phosphatidic acids (cPAs, 1-acyl-*sn*-glycerol-2,3-cyclic phosphates, Figure 1B), structurally related to LPAs. It has been proved that LPLs can activate numerous membrane receptors [2]; therefore, various species of LPLs are considered as potential therapeutic agents in different pathological states [3]. Moreover, the analogue of S1P, known as fingolimod, FTY-720 or Gilenya, has been approved, both in the USA and in European Union, as the first oral therapeutic agent for relapsing forms of multiple sclerosis [4]. This achievement has inspired further studies on medicinal chemistry and pharmacology of LPLs [5].

Although, specific cPA receptors have not been identified, numerous reports indicate that membrane LPA receptors are activated not only by LPAs but also by cPAs [6]. Moreover, cPAs are considered to be high-affinity and specific ligands of the peroxisome proliferator-activated receptor γ (PPARγ) [7]. In contrast with LPAs, cPAs are described as effective antagonists of PPARγ [8]. It seems to be even more important issue, because this nuclear receptor is involved in the regulation of adipogenesis, glucose homoeostasis and type 2 diabetes-related processes [9].

In contrast with LPAs, which are known to stimulate cell proliferation, cPAs act as antiproliferative agents and are responsible for suppression of metastasis and invasion of various cancer cells [10,11]. Since cPAs may exhibit therapeutically desired properties, many studies have been undertaken in order to synthesize metabolically stable and biologically more active cPA analogues. Derivatives of cPA, in which the *sn*-2 or *sn*-3 oxygen of the glycerol backbone was replaced by a methylene group (so called 2 carba- or 3 carba-cPA), were found to be more potent *in vitro* and *in vivo* inhibitors of melanoma cell invasion, than their unmodified counterparts [12,13]. Moreover, cPA analogues with a sulfur atom in *sn*-3 position inhibited the migration of human breast cancer MDA-MB-231 cells as effectively as did the 2 carba-cPA [14,15]. Also, our initial studies have shown the promising properties of phosphorothioate (S-cPA) and phosphorodithioate (2S-cPA) analogues of cPA (Figures 1C and 1D).

Structural studies of the mode of binding of LPA, lysophosphatidylcholine (LPC) and cPA based on crystal structures have been rather limited. Two structures of LPL-binding proteins have been solved: S1P1 receptor [16] and LPA1 receptor [17], which provide detailed information about interactions between these signalling molecules and their targets. However, there is still ambiguity about the transport of LPLs by albumin to the particular body regions and, therefore, to the exact receptor in order to trigger further effects. LPLs, as lipid derivatives, poorly dissolve in aqueous solutions. Therefore, similarly to fatty acids, they need to be bound by plasma proteins, mainly serum albumin [18], to be safely distributed within the circulatory system.

**Figure 2:**
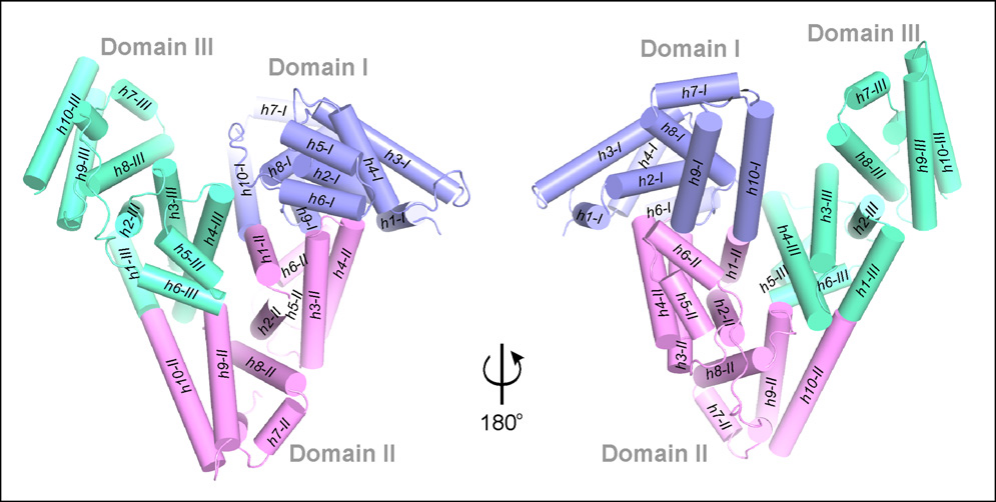
Arrangement of the helices in serum albumin The molecule is shown in two orientations for better visibility of its secondary elements. Domains I (*h1-I–h10-I*), II- (*h1-II–h10-II*) and III (*h1-III–h10-III*) are colored blue, pink and green, respectively.

Serum albumin is a three-domain helical protein (Figure 2) which binds up to seven medium or long chain fatty acids (FAs) [19]. Each domain can be further divided into two subdomains (A and B). Human serum albumin (HSA) [20] possesses two extended drug binding sites (DS1 and DS2), also called Sudlow sites [21,22], which are located in domains II and III. Albumins have a number of other binding pockets [23] responsible for the transport of small ligands, e.g. drug molecules [24] or thyroid hormones [25]. Within these pockets, serum albumin can also bind and transport LPLs. There is only one structural report about LPLs bound to HSA which was obtained in the presence of FAs [26]. This complex describes only one molecule of lysophosphatidylethanolamine (LPE) bound inside DS1. However, other studies have shown that serum albumin can bind up to five molecules of LPC [27] or three molecules of LPA [28]. Serum albumin transports also cPAs; among them the most abundant form extracted from albumin is palmitoyl-cPA (Palm-cPA, C16:0) besides myristoyl-cPA (Myr-cPA, C14:0) and stearoyl-cPA (Ste-cPA, Cl8:0), which were identified as minor albumin-bound species [29]. However, the number and location of these cPA binding sites in an albumin molecule are not known.

In this work, we initially characterized the cytotoxic properties of the novel sulfur derivatives of myristoyl-cPA, 1-*O*-myristoyl-*sn*-glycerol-2,3-cyclic phosphorothioate (Myr-S-cPA) and 1-*O*-myristoyl-*sn*-glycerol-2,3-cyclic phosphorodithioate (Myr-2S-cPA), and we present more potent analogue, Myr-2S-cPA, bound to two serum albumins HSA and equine serum albumin (ESA). The crystal structures of the investigated albumin complexes show a different number and location of Myr-2S-cPA binding sites in both proteins, indicating a species-dependent manner of cPA binding.

## MATERIALS AND METHODS

### Cell culture and MTT assay

Human prostate cancer cell lines (PC-3) were cultured in RPMI 1640 supplemented with 10% FBS, 2 mM L-glutamine and antibiotics: penicillin (100 U/ml) and streptomycin (100 μg/ml) (all from Invitrogen). Cell cultures were carried out at 37°C in a humidified atmosphere with continuous flow of 5% CO_2_. To examine cell viability the cells were trypsinized, plated into appropriate plate/dish (5x10^3^ cell/well) and allowed to grow for 24 h. After that step the media were replaced with serum-free media and the cells were starved for 24 h. Next, cells were treated with cPA analogues or LPA for 24 and 48 h (Table 1). Methanol was used as a control. After treatment with the above compounds, 25 μl of MTT solution (5 mg/ml in PBS, pH 7.4) was added into each well and left for 2 h (37°C) followed by addition of 100 μl of a lysis buffer (20% SDS w/v in 50% DMF v/v, pH 4.7). After 12 h, the absorbance of samples was measured at *λ*=570 nm using Synergy 2 plate reader. Both sulfur derivatives of cPA, Myr-S-cPA and Myr-2S-cPA were synthesized in the Institute of Technical Biochemistry, Lodz University of Technology, Poland [30].

**Table 1:** Summary of diffraction data collection and refinement statistics of HSA–Myr-2S-cPA and ESA–Myr-2S-cPA complexes Values in parentheses correspond to the last resolution shell. a *R*_merge_=Σ*_h_*Σ*_j_* | *I_hj_* − <*I_h_*> | / Σ*_h_*Σ*_j_ I_hj_*, where *I_hj_* is the intensity of observation *j* of reflection *h*. ^b^ *R*=Σ*_h_* | | *F*_o_| − | *F*_c_| | / Σ*_h_* | *F*_o_| for all reflections, where *F*_o_ and *F*_c_ are observed and calculated structure factors respectively. *R*_free_ is calculated analogously for the test reflections, randomly selected and excluded from the refinement. ^c^ ADP: atomic displacement parameter.

Structure	HSA–Myr-2S-cPA	ESA–Myr-2S-cPA
**Data collection**		
Radiation source	BESSY BL.14.2, Berlin	BESSY BL.14.2, Berlin
Wavelength (Å)	0.9184	0.9184
Temperature (K)	100	100
Space group	*P*1	*P*6_1_
Unit cell parameters		
*a*, *b*, *c* (Å)	37.9, 85.2, 96.3	93.9, 93.9, 141.8
*α*, *β*, *γ* (°)	105.5, 89.8, 101.3	90.0, 90.0, 120.0
Oscillation range (°)	0.3	0.4
Resolution (Å)	46.35–2.26 (2.40–2.26)	50–2.48 (2.58–2.48)
Reflections collected/unique	98215/49652	115255/24973
Completeness (%)	93.5 (90.1)	99.6 (99.4)
Multiplicity	2.0	4.6
*R*_merge_a (%)	5.0 (36.9)	8.8 (88.1)
<*I*/σ(*I*)>	12.4 (2.3)	14.5 (2.1)
**Refinement**		
*R*_free_ reflections	1012	1012
No. of atoms (non-H) protein/ligand/solvent	9274/195/310	4575/57/99
*R*_work_/*R*_free_^b^ (%)	20.7 (25.3)	17.3 (22.9)
Mean ADP^c^ (Å^2^)	46.2	55.7
R.M.S.D. from ideal geometry		
Bond lengths (Å)	0.019	0.020
Bond angles (^o^)	1.9	1.8
Ramachandran statistics (%)		
Favoured/allowed/outliers	95/5/0	96/4/0
PDB code	5ID7	5ID9

### Crystallization of HSA and ESA complexes with Myr-2S-cPA

Fatty acid free HSA at 99% purity (Sigma–Aldrich) and lyophilized ESA with the purity no less than 96% (Equitech-Bio) were additionally purified in a two-step procedure [31]. The complexes HSA–Myr-2S-cPA and ESA–Myr-2S-cPA were formed by incubation of the respective albumins with a 10-molar excess of Myr-2S-cPA, with constant mixing overnight at room temperature. Prior to incubation, the mixtures were heated to 50°C for approximately 1 h. Both samples were centrifuged before the crystallization setup. The crystallization conditions previously established for HSA [24] and ESA [31] were optimized to get crystals of their complexes with Myr-2S-cPA. The best crystals were grown by the hanging drop vapour diffusion method against the solutions containing: 28% PEG3350, 50 mM phosphate buffer at pH 7.5 for HSA–Myr-2S-cPA, or 1.8 M ammonium sulfate, 0.1 M acetate buffer at pH 4.5 for ESA–Myr-2S-cPA. The concentration of albumin used for crystallization was 2 mM for HSA and 1.2 mM for ESA.

### Diffraction data collection and structure determination

Diffraction data collection was carried out at 100 K with a single crystal of each complex, using oscillation method on the synchrotron beam line 14.2 in Berlin, Germany [32]. A crystal of HSA–Myr-2S-cPA was directly flash frozen without additional cryoprotection, whereas a crystal of ESA–Myr-2S-cPA was transferred to a cryoprotecting solution of 70% Tacsimate (Hampton Research) at pH 6.0 before diffraction experiment [33]. Both diffraction data sets were processed with *XDS* [34] and *XSCALE* [35] to the resolution of 2.48 Å (1 Å=0.1 nm) (ESA–Myr-2S-cPA) and 2.26 Å (HSA–Myr-2S-cPA). In the case of HSA–Myr-2S-cPA, *XDSAPP* graphical user interface was used [36]. HSA–Myr-2S-cPA structure was solved by molecular replacement with the starting model of HSA (PDB ID: 3A73) [37] using *Phaser* [38]. The rigid body procedure of *REFMAC* [39] was used to determine the ESA–Myr-2S-cPA complex with the structure of ESA complexed with naproxen (Nps, PDB ID: 4OT2) as a model [40]. All ligands and water molecules were deleted from both models before structure determination. At a later stage of refinement, TLS parameters [41,42] were introduced. The atomic coordinate and geometry libraries of the ligand were generated in *JLigand* [43] from the *CCP4* package [44]. The ligands were manually fitted into the electron density using *Coot* [45] and both models were refined with *REFMAC* [39]. The quality of both structures was controlled by *R, R_free_* [46] and other geometrical parameters. The programs *PROCHECK* [47] and *MolProbity* [48] were used for evaluation of the final models. A summary of data collection and refinement statistics are given in Table 1. Both structures were deposited in Protein Data Bank (PDB) [49] with accession numbers: 5ID7 (HSA–Myr-2S-cPA) and 5ID9 (ESA–Myr-2S-cPA).

## RESULTS AND DISCUSSION

### Inhibition of the growth and proliferation of prostate cancer cells (PC-3) by Myr-2S-cPA

**Figure 3:**
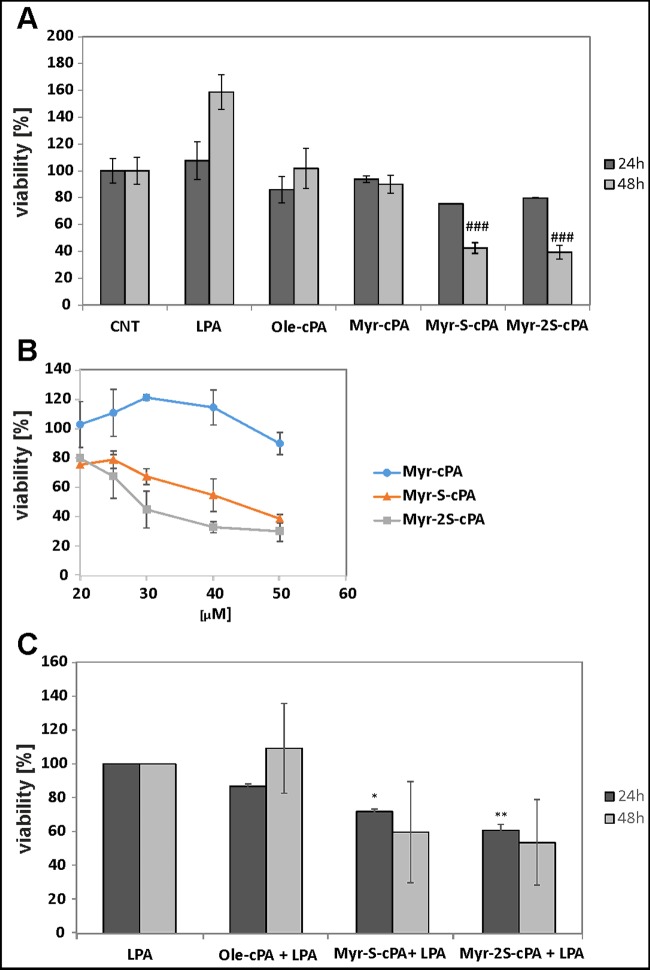
Effects of LPA and cPA analogues on the viability of PC-3 cells (**A**) Effects of cPA, cPA analogues (20 μM) and LPA (10 μM) on PC-3 cells viability after 24 or 48 h incubation; ###, significantly different from methanol-treated cells (CNT) (P < 0.001). (**B**) Effects of cPA analogs concentration on PC-3 cell viability after 24 h; IC50 was extrapolated from the inhibition curve. (**C**) Effects of cPA analogues on LPA-dependent proliferation of PC-3 cells; cell viability was tested after 24 or 48 h incubation with 20 μM cPA or its analogues in the presence of 10 μM LPA; results significantly different from cells treated only with LPA were marked with *, *P* < 0.05 and **, *P* < 0.01.

To assess the cytotoxic potential of Myr-2S-cPA, its influence on the viability of PC-3 cancer cells was analysed (Figure 3). Naturally occurring cPA with oleoyl (Ole-cPA) or myristoyl (Myr-cPA) fatty acid chains were not toxic to the cells at 20 μM concentration. A cytotoxic effect was observed for Ole-cPA at 50 μM (39%), whereas Myr-cPA at the same concentration moderately reduced PC-3 cell viability (11.2% ±7.6). Replacement of phosphate by phosphorothioate or phosphorodithioate group dramatically increased the cytotoxic activity of cPA with myristoyl fatty acid residue. Thus, Myr-S-cPA at 20 μM decreased the number of viable cells by more than 24% after 24 h and by approximately 57% after 48 h of treatment (Figure 3A). Similarly, Myr-2S-cPA at 20 μM reduced PC-3 cell viability more than 20% after 24 h and 60% after 48 h. A detailed analysis indicated that Myr-S-cPA and Myr-2S-cPA reduced cell viability in a dose-dependent manner and at 50 μM concentration decreased PC-3 cell viability by around 61% and 70% respectively (Figure 3B). The IC_50_ value determined for Myr-2S-cPA after 24 h incubation was 29.0 μM, whereas for Myr-S-cPA it was 42.8 μM.

In contrast with the cPA analogues, 10 μM LPA stimulated proliferation of PC-3 (Figure 3A) and this compound was used to determine the antiproliferative potential of Myr-S-cPA and Myr-2S-cPA. Despite of the presence of LPA in the culture media, both compounds reduced the number of cells by approximately 28% and 39% after 24 h respectively. Forty-eight hours of treatment with the compounds decreased LPA-dependent growth of PC-3 cell to 60% and 54% (Figure 3C).

Of course, biological activities of different LPLs are not only dependent on the structure of their polar head group (in this case, their cyclic moiety). The other important factor is the nature of the acyl chain, i.e. its length, saturation or additional modifications. However, it should be noted that the data describing the best documented activities of cPA [50] or LPC [51] species are available for only 18:1 (oleoyl), 16:0 (palmitoyl) and 18:0 (stearoyl) forms. Although molecular mechanisms responsible for cPA activities are not known well, numerous reports indicate that different cPA species can activate membrane LPA receptors. In our study, we intentionally investigated cPAs with shorter acyl chains, which in their size are more similar to S1P. In that case, the cytotoxic effects of Myr-S-cPA and Myr-2S-cPA may result not only from the interactions with LPA receptors, but also from the interactions with the S1P receptors, as it has been previously suggested [52].

### Binding of Myr-2S-cPA to HSA

**Figure 4:**
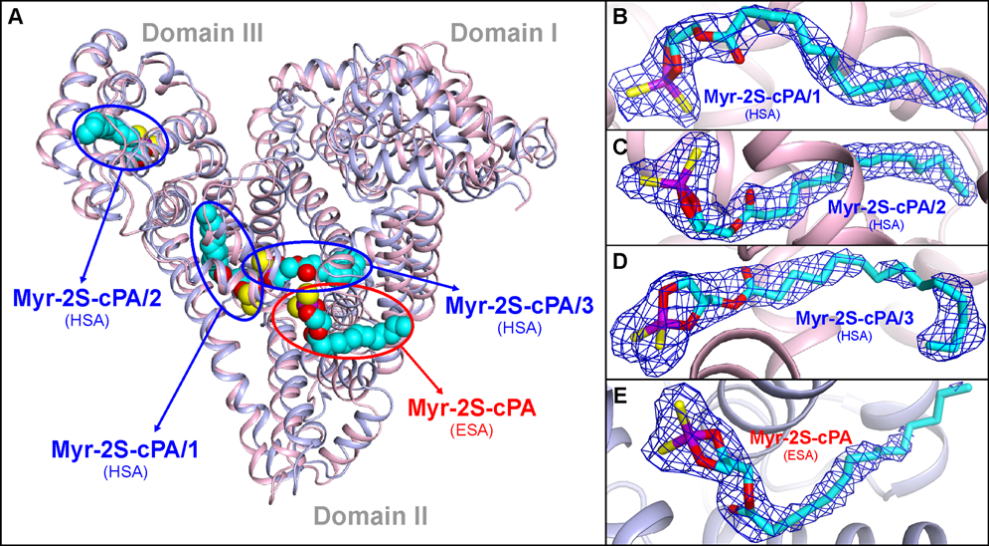
Myr-2S-cPA binding sites identified in HSA and ESA (**A**) Superposition of HSA (light pink) and ESA (light blue) complexes with Myr-2S-cPA. (**B**) Conformation of the Myr-2S-cPA/1 in site 1 of HSA. (**C**) Conformation of the Myr-2S-cPA/2 in site 2 of HSA. (**D**) Conformation of the Myr-2S-cPA/3 in site 3 of HSA. (**E**) Conformation of the Myr-2S-cPA bound in ESA. 2Fo–Fc electron density maps for the bound ligands are contoured at 1 σ level. Atoms of the Myr-2S-cPA are colored as follows: carbon (cyan), oxygen (red), sulfur (yellow) and phosphorus (violet); the color coding is consistent in all figures.

The structure of HSA–Myr-2S-cPA, determined at the resolution of 2.26 Å in the *P*1 space group with two monomers of HSA in the asymmetric unit, reveals three unique binding sites of Myr-2S-cPA in each monomer (Figure 4A). Bound Myr-2S-cPA in each site of HSA is referred as Myr-2S-cPA/1, Myr-2S-cPA/2 and Myr-2S-cPA/3. Conformation of the bound ligands in the corresponding Myr-2S-cPA binding locations is very similar in both chains, thus we will further refer in detail only to monomer A (unless stated otherwise). The identified binding sites of Myr-2S-cPA in HSA are: Drug Site 2 (DS2) of subdomain IIIA, fatty acid site 5 (FA5) of subdomain IIIB and the chamber created at the interface between domains II and III, which is formed in the elongation of Drug Site I (DS1) of subdomain IIA. Despite significant flexibility of the ligand, the sharp and well-defined electron density allowed unambiguous determination of the ligand conformation in all binding locations, especially regarding the polar fragment of Myr-2S-cPA (Figures 4B, 4C and 4D).

**Figure 5:**
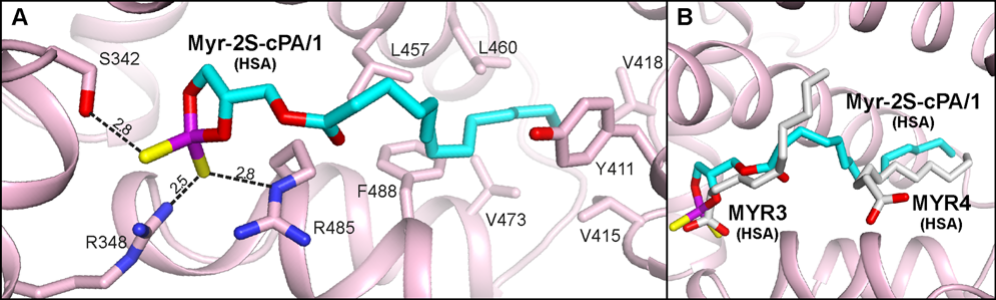
Myr-2S-cPA/1 binding site of HSA (**A**) Amino acids residues involved in the interactions with ligand via hydrogen bonds [Å]. (**B**) Comparison of the Myr-2S-cPA/1 (cyan) binding site with the same pocket of HSA (PDB ID: 1E7G) in which two myristic acid molecules (gray) are bound.

Myr-2S-cPA/1 is located in the DS2 pocket, a typical binding site for more bulky molecules. The electrostatic interactions involve both sulfur atoms of the ligand, which are hydrogen bonded with the side chains of Ser^342^, Arg^348^ and Arg^485^ (Figure 5A). The aliphatic chain of Myr-2S-cPA/1 spreads across the big central chamber and anchors in the hydrophobic part of DS2. This cavity is built by the following residues from the region *h2-III*–*h6-III*: Phe^488^, Val^473^, Val^415^, Val^418^, Leu^423^, Val^426^, Leu^460^, Leu^430^, Leu^453^, Val^456^, and also by the side chains of Tyr^411^ and Lys^414^. The aliphatic tail of Myr-2S-cPA/1 presents several van der Waals contacts with residues in this compartment (Figure 5A). The rear part of the central chamber of DS2 remain filled with solvent, thus the ligand may be bound more loosely and the little less clear electron density for the central portion of Myr-2S-cPA/1 can be explained.

The location of the Myr-2S-cPA/1 is overlapping with two binding sites of fatty acids–FA3 and FA4 [19]. The cyclic polar head of Myr-2S-cPA/1 is facing towards the cluster of polar residues of HSA–Ser^342^, Arg^348^ from *h9-II*, and Arg^485^ from *h6-III*. These residues are also responsible for the interactions with the carboxyl groups of FA bound in the FA3 site. Thus, after superposition of the structure of HSA complexed with FA (PDB ID: 1E7G) with the complex presented here, the positions of the polar heads of Myr-2S-cPA/1 and FA overlap well (Figure 5B). On the other hand, the conformation of the apolar fragment of the ligand shows significant similarity with the other FA molecule, which is bound within FA4. However, in this case, the carboxyl group of FA is pointing towards the entrance of DS2. The pocket of subdomain IIIA is also the major binding site of aromatic drug molecules in HSA (e.g. diflunisal, diazepam, ibuprofen, propofol) [24,53] and ESA (diclofenac, Nps) [54]. Moreover, this site is also responsible for thyroxin binding in HSA [25].

**Figure 6:**
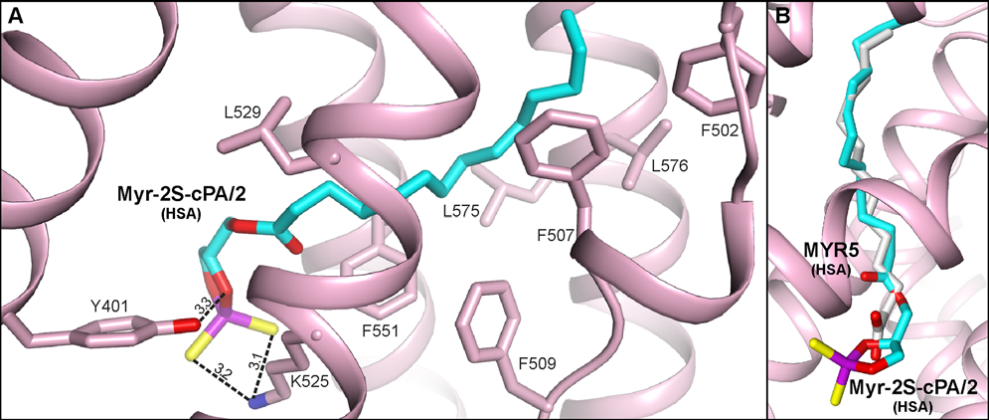
Myr-2S-cPA/2 binding site of HSA (**A**) Amino acids residues involved in the interactions with ligand via hydrogen bonds [Å]. (**B**) Comparison of the Myr-2S-cPA/2 binding site with the same pocket of HSA (PDB ID: 1E7G) where the myristic acid residue (gray) is bound.

The FA5 pocket is a long and narrow chamber, which is almost entirely built of hydrophobic residues placed in the centre of a helical bundle (*h7-III*–*h10-III*) of the subdomain IIIB. Only the closest environment of the entrance presents polar character with a predominance of positively charged residues. The aliphatic chain of Myr-2S-cPA/2 is anchored at the bottom of the FA5 via the van der Waals contacts with the side chains of Phe^502^, Phe^507^, Phe^509^, Leu^532^, Phe^551^, Leu^575^, Val^576^, and extends up to the entrance, where the polar head of the ligand is placed. The cyclic head of Myr-2S-cPA/2 is oriented alongside the side chain of Lys^525^ and forms two hydrogen bonds between its sulfur atoms and Nζ of Lys^525^ (Figure 6A). Additionally, a hydrogen bond is observed between oxygen from *sn*-2 position of glycerol moiety of Myr-2S-cPA/2 and hydroxyl group of Tyr^401^ from *h2-III* with a distance of 3.3 Å. The conformation of Myr-2S-cPA/2 is in agreement with the FAs bound inside this pocket of HSA (Figure 6B) [19] where the carboxyl group of FAs also protrudes outside the pocket. Due to the elongated shape, the FA5 pocket primarily binds lipidic molecules [19]. However, the pocket was also identified in HSA as the binding site of thyroxine [5] and propofol [53]. Two other ligands, fusidic acid [55] and oxyphenbutazone [24], bind in a close proximity to the FA5 entrance.

The Myr-2S-cPA/3 is bound in the site located at the interface of domains II and III, in the centre of serum albumin molecule. The binding chamber, which is actually the elongation of DS1 of subdomain IIA, is created as a result of the movement of several albumin components, i.e. an analogous domain rearrangement of HSA to those observed in complexes with FAs [24]. The terminal part of the aliphatic tail of Myr-2S-cPA/3 is placed in the central compartment of DS1 of subdomain IIA; however its central part and the polar head, after the passage between Trp^214^ and Phe^211^, crosses the normal boundaries of DS1 through a narrow tunnel to reach an additional chamber. The tunnel connects with DS1 as a result of the movement of the side chain of Trp^214^ together with a shift of the inter-domain helix *h20-I–h1-II*. It is worth noting that this additional distant compartment of DS1 is in a very close neighbourhood to the interface of subdomain IIIA, walled by the residues from *h6-III* and *h4-III* and thus close to the DS2, i.e. just approximately 6 Å outside the binding site of Myr-2S-cPA/1 on the other site of the residues.

**Figure 7:**
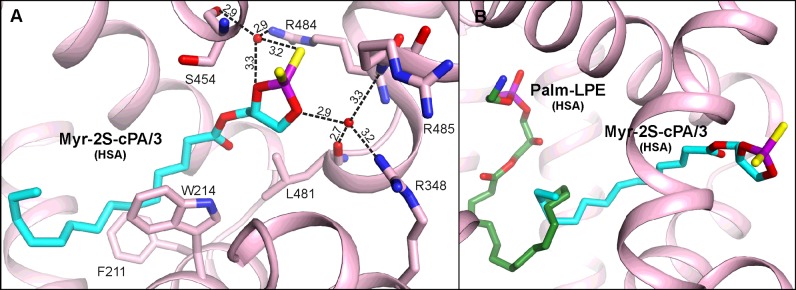
Myr-2S-cPA/3 binding site of HSA (**A**) Amino acids residues interacting with ligand via hydrogen bonds [Å]. (**B**) Comparison of the Myr-2S-cPA/3 binding site with the place of Palm-LPE (green) bound in HSA (PDB ID: 3CX9).

Myr-2S-cPA/3 is not directly hydrogen bonded to the protein; however, there are water-mediated bonds created between the ligand and two waters (Figure 7A). One is created by the *sn*-3 glycerol oxygen of Myr-2S-cPA/3, which further interacts via the water (2.9 Å) with the carbonyl oxygen of Leu^481^, the main chain nitrogen of Arg^485^ from *h6-III*, and the nitrogen from the guanidyl group of Arg^348^ from *h9-II*. The other bonds, which involve water, are created via one of the sulfur atoms of Myr-2S-cPA/3 (3.2 Å) and the *sn*-2 oxygen of Myr-2S-cPA/3 glycerol moiety (3.3 Å). This water molecule is hydrogen-bonded with the side chain of Arg^484^ (2.9 Å) from *h6-III* and with the carbonyl oxygen of Ser^454^ (2.9 Å) from *h4-III*. Such water-mediated contacts between ligand and protein are quite often observed in structures of macromolecules possessing large binding pockets, including albumins. Additionally, these contacts provide more flexibility in interactions with protein for ligands lacking proton donors and rich in proton accepting groups.

Although Myr-2S-cPA/3 binding site partially belongs to the DS1 pocket, which has been described as the binding site of LPE with palmitoyl residue (Palm-LPE) (PDB ID: 3CX9) [26], the conformation of these molecules indicates a different binding mode (Figure 7B) and both sites should rather be treated as separate LPLs binding sites (discussed below in more detail). The same situation is observed with FA7 bound in DS1 [19]. There are many ligands, which bind in DS1 of subdomain IIA. However, the vast majority is bound in the main compartment of DS1, i.e. in the region where aliphatic tail of Myr-2S-cPA is bound [24]. Only a few ligands bind much closer to the central part of Myr-2S-cPA; these are: indomethacin [24], iodipamide [24], diclofenac [56] and some of the fluorescent probes [57,58]. However, none of the ligands occupy the compartment where the cyclic head of Myr-2S-cPA is bound.

As mentioned before, the electron density maps 2*F*_o_*−F*_c_ for all binding sites of Myr-2S-cPA determined in the HSA complex are well defined for the polar fragment of the bound ligands. However, strong hydrophobic character and the extensive mobility of the myristoyl moiety of Myr-2S-cPA are the reasons of a little less clear electron densities in some ligand regions. This is the case of the central fragment of Myr-2S-cPA/1 and for the terminal part of Myr-2S-cPA/3. Inside the DS2 pocket of HSA, both ends of Myr-2S-cPA/1 are deeply anchored inside the pocket; however, in the central part of the ligand, some conformational freedom is observed, mostly due to the lack of tight interactions between Myr-2S-cPA/1 and the serum albumin side chains in the large main chamber of DS2, where the ligand presents no significant interactions with protein. An analogous situation is observed for a fragment of Myr-2S-cPA/3, where it is placed in a big compartment of DS1. In the case of the Myr-2S-cPA/2 location in HSA the conformation of the fatty acid tail Myr-2S-cPA/2 is unquestionable, mostly due to the tight hydrophobic interactions between the protein and Myr-2S-cPA/2 presented in the narrow FA5 pocket.

### Binding of Myr-2S-cPA to ESA

**Figure 8:**
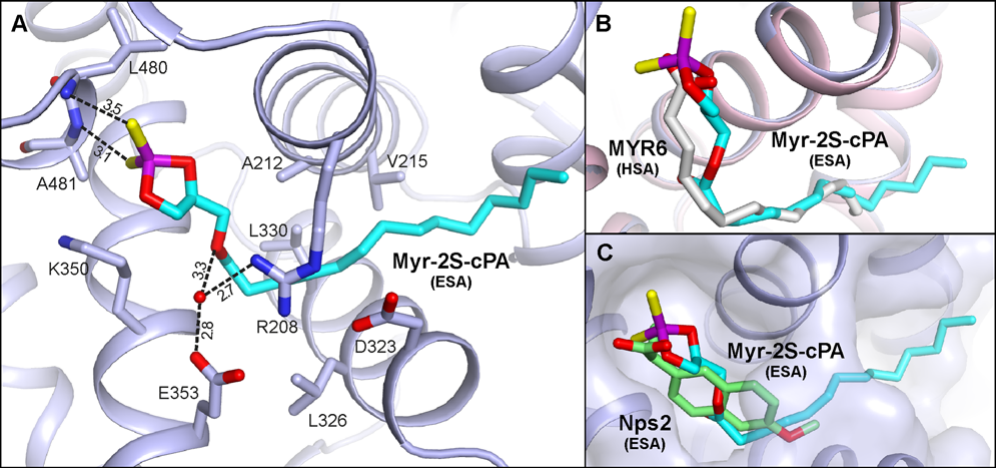
Myr-2S-cPA binding site of ESA (**A**) Amino acids residues interacting with ligand via hydrogen bonds [Å]. (**B**) Comparison of the Myr-2S-cPA binding site in ESA (light blue) with the analogical pocket in HSA where myristic acid (gray) is bound (light pink; PDB ID: 1E7G).

The structure of the ESA–Myr-2S-cPA complex, determined to the resolution of 2.48 Å in *P*6_1_ space group with a single monomer in the asymmetric unit, includes only one binding site of Myr-2S-cPA (Figure 4A). This binding site is located in the cleft created between helices *h2-II*, *h3-II* of subdomain IIA, and helices *h8-II, h9-II* of subdomain IIB, which is formed also with a contribution of the helices *h5-III*, *h6-III* of subdomain IIIA and the short loop between them. The niche was recognized in earlier studies of ESA complex with Nps as the lower affinity binding site for the ligand [40,54] and the site of antihistamine drug, cetirizine [59]. In HSA studies, FA6 pocket has been identified as the secondary site of other NSAIDs, ibuprofen and diflunisal [24], the anaesthetic, halothane [53], some fluorescent probes [58,60] and perfluorooctane [61]. The polar cyclic moiety of Myr-2S-cPA is located in the same compartment as the Nps molecule (Figure 8C) in the structure of ESA–Nps complex (PDB ID: 4OT2) alongside Lys^350^. Both sulfur atoms of Myr-2S-cPA are involved in the interactions with residues of subdomain IIIA (Figure 8A); they create hydrogen bonds with the backbone nitrogen atoms of Leu^480^ (3.5 Å) and Ala^481^ (3.1 Å). Additionally, the *sn*-1 oxygen of Myr-2S-cPA forms a water-mediated hydrogen bond (3.3 Å) with Glu^353^ and Arg^208^ side chains. The latter residues, together with Asp^323^, create a closed ‘buckle’ over the binding pocket which covers the aliphatic fragment of the ligand. Myr-2S-cPA presents several van der Waals interactions with hydrophobic residues of albumin: Ala^212^, Val^215^, Leu^326^, Leu^330^, Gly^327^, as well as with the above-mentioned ‘buckle’ (Figure 8A). The last four-carbon fragment of Myr-2S-cPA tail is protruding outside the pocket, into the solvent region. The conformation of the ten-carbon fragment of the myristoyl moiety of Myr-2S-cPA is well-defined in the electron density (Figure 4E). Thus, poorer electron density around the terminal fragment is understandable, due to the lack of interactions between the ligand and the protein. This binding pocket was also identified in HSA studies as FA6 [19] and, comparing the conformation of Myr-2S-cPA and FA in this cavity, they present an analogous conformation (Figure 8B).

### Comparison of Myr-2S-cPA binding to HSA and ESA

Although serum albumins isolated from different species are highly homologous, their binding properties cannot be strictly treated as such. A comparison of both albumin complexes with Myr-2S-cPA shows significant differences in the ligand binding mode. None of the three binding pockets of Myr-2S-cPA described in HSA overlaps with the single site in ESA, although both serum albumins show quite big sequence identity (76%) and structural homology (2.61 Å R.M.S.D. for Cα superposition). The superposed structures (Figure 4A) present a different orientation of the domains, especially domain I, and show substantial shape differences of their binding cavities.

In the case of the DS2 binding pocket, identified as Myr-2S-cPA/1 site in HSA, difference of its architecture between both albumins is mostly a consequence of the above-mentioned domain rearrangement in both proteins. In ESA, a bend of the helix *h4-III* and its movement of approximately 3 Å, starting from its middle section, lead to a significant shift of several side chains. This shift results in the placement of the side chain of Glu^449^ in a close neighbourhood of Arg^437^ and Arg^484^ (note that the sequence positioning in ESA is different for the residue range Lys^116^–Ala^583^, due to the single deletion after Leu^115^), where it creates a network of hydrogen bonds with the polar cluster responsible for binding of Myr-2S-cPA/1 in HSA (see section ‘Binding of Myr-2S-cPA to HSA’). The shifted glutamate residue precludes Myr-2S-cPA recognition and thus binding within DS2 of ESA. Additionally, a different structural arrangement within subdomain IIIA in ESA results in narrowed DS2 pocket in a region where the aliphatic chain of Myr-2S-cPA/1 is bound in HSA, which is visible especially in the neighbourhood of Tyr^411^.

The binding pocket of Myr-2S-cPA/2 in HSA has several different amino acids at the entrance in comparison with ESA. These differences concern substitutions: Met^458^ to Leu^457^ (the first residue refers to HSA; this convention is preserved for the whole section), Lys^402^ to Asp^401^, Ala^502^ to Ser^501^, Asp^549^ to Gly^458^, Arg^521^ to Lys^520^. Although these residues are not directly involved in Myr-2S-cPA/2 binding, they influence the electrostatic character of the pocket entrance in both proteins. This, together with conformational rotation of Phe^550^ in ESA, makes the pocket inaccessible for Myr-2S-cPA in ESA.

Shape and size of the Myr-2S-cPA/3 binding pocket in HSA seem to be highly dependent on the conformational rearrangements of albumin domains. This place is also highly dissimilar in both albumin species. Unlike the previously compared Myr-2S-cPA sites, in Myr-2S-cPA/3 site, there are several amino acid substitutions in a close neighbourhood of the ligand: Val^344^ to Ser^343^, Ser^454^ to Ala^453^, Val^455^ to Leu^454^, Phe^211^ to Val^210^ and Ala^215^ to Ser^214^. In DS1 of ESA, this site is not fully opened to the additional chamber where the cyclic head of Myr-2S-cPA/3 could be incorporated. Therefore, this compartment of DS1 in ESA is too small to bind such a large ligand.

The FA6 pocket, which is recognized as the only one Myr-2S-cPA binding site in ESA, presents very similar architecture in both, ESA and HSA; however, the HSA complex shows a bound fragment of polyethylene glycol, which was absorbed from the crystallization solution. There is a possible scenario which promotes Myr-2S-cPA binding in FA6 of ESA. Most probably, Val^482^ to Ala^481^ substitution and thus, the presence of a residue with reduced size of side chain, increases the availability of backbone nitrogen atoms, placed at the initial section of the helix (Leu^480^ and Ala^481^), for the creation of hydrogen bonds with negatively charged molecules in this compartment. Additionally, another Asn^483^ to Glu^482^ substitution leads in ESA to the rotation of the side chain of Ser^480^ and affects the closest environment of the binding site.

Both complexes discussed here were crystallized in different conditions and to diminish, as much as possible, the influence of the crystallization components on Myr-2S-cPA binding, we have performed the complexation step before crystallization, suspending both proteins in the same buffer. It is possible that different crystallization pH could affect, to a certain extent, the binding properties of ESA and HSA especially in the FA6 site. This pocket is exposed to the solvent and its binding ability may be altered in a different pH. This should not be the case of the other Myr-2S-cPA binding sites (DS2, FA5 and the extended chamber of DS1) found in HSA. The steric limitations, caused by different amino acids side chains conformation of ESA pockets in comparison with HSA, prevent Myr-2S-cPA binding in ESA in the same locations. Hypothetically, Myr-2S-cPA bound in the same place in ESA and with similar conformation as it is bound in HSA, would create severe steric clashes. The other factors, as pH or crystallization components, at this point, have rather minor influence on Myr-2S-cPA binding. The crystal structures of ESA, obtained in different crystallization conditions, e.g. PDB ID: 4F5U (pH 6.0), 5IIU (pH 6.9) or 5HOZ (pH 9.0), present almost identical arrangement of side chains within DS1, DS2 and FA5 pockets. Thus, we could postulate that the differences between HSA and ESA binding properties of Myr-2S-cPA are influenced more by the individual characteristics of the albumins than by pH of crystallization buffer. Although, both studied structures were crystallized from altered conditions and were solved in different space groups, the binding pockets are not involved in the crystal contacts of albumin molecules with symmetry related monomers.

### LPLs interactions with albumin

It has been shown that some LPLs possess certain activities only in the albumin-bound form. To list only a few examples, LPA, transported by albumin, is responsible for the stress fibre formation [62]. Moreover, by the interactions with LPC, serum albumin increases the availability of LPCs for their receptors [27,51,63–65]. Activation of osteoblast development during bone repair process is linked with the action of LPAs bound to albumin [66]. Serum albumin, through LPLs binding, lowers their free fraction in blood; however, these molecules bound by albumin are protected from enzymatic degradation [67]. Also in the presence of FAs, serum albumin exhibits lower affinity to LPE, which suggests that FAs may act as regulators of the extracellular LPLs activity [26].

Only one structure of HSA with LPL molecule, other than Myr-2S-cPA presented in this work, is found in the PDB. The structure of Palm-LPE (C16:0) was obtained in the presence of myristic acid (PDB ID: 3CX9) [26]. In that complex, one Palm-LPE binding site is described; it is the DS1 of subdomain IIA. Palm-LPE presents a bent conformation, with its hydrophilic head anchored in the polar region of the central chamber of DS1 and with the aliphatic tail curved in the hydrophobic section of the pocket. Only a terminal fragment of the fatty acid tail of Palm-LPE runs in a close distance to the Trp^214^, the same compartment where the terminal fragment of Myr-2S-cPA/3 is placed. However, binding locations of Myr-2S-cPA/3 and Palm-LPE should not be considered as the same binding sites (Figure 7B). Palm-LPE presents several hydrogen bonds within main compartment of DS1. Palm-LPE interacts with the side chains of Glu^153^, Gln^196^, Arg^257^ and His^242^. The different structural architecture of the hydrophilic head of both LPLs causes the alteration in the ligand binding mode. The cyclic moiety of Myr-2S-cPA is much more bulky than the elongated Palm-LPE, and it presents much less conformational latitude caused by the additional bond with the phosphate moiety. This gives no possibility to fit the cyclic phosphorodithioate moiety in the narrow compartment around Arg^257^. Additionally, in ESA, as a consequence of differently rotated domain I, the side chain of Tyr^149^ is shifted to the compartment of DS1 and overlaps with the superposed Palm-LPE residue.

## CONCLUSION

The crystal structures of HSA and ESA in complexes with Myr-2S-cPA prove that cPA can be transported by albumins. cPAs, as bioactive compounds structurally related to the corresponding LPAs, are promising molecules for drug development. We have shown that phosphorothioate and phosphorodithioate derivatives of cPAs, derived from myristic acid, in which one or two of the non-bridging oxygen atoms in the phosphate group were replaced with a sulfur atoms, exhibit enhanced cytotoxic activity. The substitution of the phosphate group of Myr-cPA with the sulfur atoms increased the biological action of the corresponding derivatives, Myr-S-cPA and Myr-2S-cPA. These compounds reduced PC-3 cell viability up to 60% in comparison with the unmodified LPA and cPA. However, Myr-2S-cPA seems to be more potent than the single substituted phosphorothioate cPA, with the IC_50_ value of 29.0 μM after 24 h incubation, which is almost 30% lower than the IC_50_ of Myr-S-cPA. The other factors which may alter the biological activity of cPA derivatives are modifications of the aliphatic tail length or its saturation. Compounds with shorter aliphatic chain may imitate the ligands of S1P receptors and potentially cause increased biological activity.

Crystal structures of HSA and ESA complexes with Myr-2S-cPA shed a new light on the role of albumin in the transport of cPAs, important signalling molecules. Both proteins present highly species-dependent binding manner of Myr-2S-cPA. HSA binds three molecules of Myr-2S-cPA, inside of the DS2 and FA5 pockets, and within an enlarged compartment of DS1, whereas ESA possesses only one binding site of this ligand in the FA6 pocket. This is not very surprising, since our previous studies have shown differences in the binding of the same ligand by albumins from different species [40,68].

An interesting finding is that there is no analogy to the previously reported HSA–Palm-LPE complex in the location of the bound ligands. The Palm-LPE is bound only in a central subchamber of DS1, located in subdomain IIA of HSA [26]. Apart from Palm-LPE, the HSA–Palm-LPE structure includes five bound molecules of myristic acid, which occupy the FA2–FA6 sites in an analogous manner to the FAs bound in HSA–FAs complexes [19]. It is worth noting that three of the myristates overlap with Myr-2S-cPA bound to HSA in the reported HSA–Myr-2S-cPA complex; FA3 and FA4 sites correspond to the Myr-2S-cPA/1 site and FA5 pocket corresponds to Myr-2S-cPA/2 binding site. The binding pocket of Myr-2S-cPA, identified in ESA, is also analogous to the FA6 localization in HSA–Palm-LPE. It cannot be excluded that HSA should be able to transport more than one Palm-LPE under conditions, which introduce fewer FAs.

We have shown that albumin binding of LPLs not only can be different in respect to various LPLs, but also can vary between albumin species. Therefore, cautious interpretation of the results should be implemented not only when albumins of different species are utilized in the experimental environment *in vitro*, but also when different organisms are taken under investigation during the experiments *in vivo*. Moreover, the use of insufficiently purified albumin, with residual fatty acids remaining bound inside the binding pockets, may also introduce bias to the experiments and provide misleading results.

## Abbreviations

cPA: cyclic phosphatidic acid
DS: drug site
ESA: equine serum albumin
FA: fatty acid
HSA: human serum albumin
LPA: lysophosphatidic acid
LPC: lysophosphatidylcholine
LPE: lysophosphatidylethanolamine
LPL: lysophospholipid
Myr-S-cPA: 1-*O*-myristoyl-*sn*-glycerol-2,3-cyclic phosphorothioate
Myr-2S-cPA: 1-*O*-myristoyl-*sn*-glycerol-2,3-cyclic phosphorodithioate
Nps: naproxen
Palm: palmitoyl
PC: prostate cancer
PDB: Protein Data Bank
PPARγ: peroxisome proliferator-activated receptor γ
S1P: sphingosine-1-phosphate
